# Polarization-Insensitive Lithium Niobate-on-Insulator Interferometer

**DOI:** 10.3390/mi15080983

**Published:** 2024-07-30

**Authors:** Jiali Liao, Linke Liu, Yanling Sun, Zihao Wang, Wei Li, Jinrong Lan, Lin Ma, Zhenzhong Lu

**Affiliations:** 1 School of Optoelectronic Engineering, Xidian University, Xi’an 710071, China; 22191215011@stu.xidian.edu.cn (L.L.); wangzihao@xidian.edu.cn (Z.W.); wli0805@stu.xidian.edu.cn (W.L.); 21051212315@stu.xidian.edu.cn (J.L.); lma@mail.xidian.edu.cn (L.M.); zzluxidian@126.com (Z.L.); 2 Science and Technology on Electromechanical Dynamic Control Laboratory, Xi’an 710065, China

**Keywords:** electro-optic modulation, polarization independent, lithium niobate, mode converter

## Abstract

The key components of a polarization-independent electro-optic (EO) interferometer, including the beam splitter, mode converter, and directional coupler, are designed based on a lithium niobate (LN) platform on an integrated insulator to ensure high extinction ratios. By elaborately designing the geometric structure of the multimode interference (MMI) coupler, beam splitting of equal proportions and directional coupling of higher-order modes are realized. The most prominent characteristic of the proposed interferometer is polarization insensitivity, which is realized by converting TM polarization into TE polarization using a mode converter, providing conditions for the study of light with different polarizations. At 1550 nm, the visibility of the interferometer is 97.59% and 98.16% for TE and TM, respectively, demonstrating the high performance of the proposed LN polarization-independent interferometer.

## 1. Introduction

Over the past decade, there has been a series of significant research progresses in optical interconnections, which have gradually replaced copper interconnections due to their superior bandwidth [1,2,3,4,5]. One crucial component in this transmission is the EO modulator, responsible for converting electrical signals into optical signals [6,7,8]. Although many silicon EO modulators have been reported, it is believed that their current capabilities are approaching their physical limits, such as the modulation speed and optical loss, while future applications will require much higher performance for modulators.

Despite advancements in various materials [9,10,11,12,13], LN remains the preferred choice for EO modulators due to its remarkable linear EO effect, substantial EO coefficient, low optical absorption loss, and proven reliability [14,15]. Recently, the development of lithium niobate thin film on insulators has further improved LN modulators by offering a higher refractive-index contrast compared to traditional bulky LN modulators. One limitation of LN modulators is their polarization dependence, which results from the anisotropy of LN crystals. However, polarization-independent modulators are highly desired by many application fields, such as optical communication [4,16,17,18,19], optical signal processing, and microwave photonics [20,21]. A polarization-independent EO modulator with a loss of 0.04 dB was reported, but this modulator used Z-CUT LN, which could not use the high EO coefficient of X-CUT LN; therefore, the modulator needed high voltage [22]. An LN interferometer, which realized a polarization beam splitter and a polarization-insensitive switch by LN thermo-optic MZI, was reported in [23].

In this paper, we proposed a novel polarization-independent LN interferometer by designing a mode converter to convert TM to TE polarization before the EO modulation. The interferometer was designed based on an X-CUT LN crystal, which enabled effective modulation along the *x*-axis, leveraging the substantial EO coefficient of LN in this direction and aligning the crystal polarization with the light field in the waveguide. Apart from the mode converter, the functional components of the interferometer were well designed, including the LN waveguides and the 1 × 2 and 2 × 2 MMI. The performance of the designed polarization-independent interferometer was investigated by simulation. Despite the polarization, the input light was modulated with high extinction ratios, demonstrating the superior polarization independence of the proposed LN interferometer.

## 2. Design of the Polarization-Independent LN EO Interferometer

**Figure 1:**
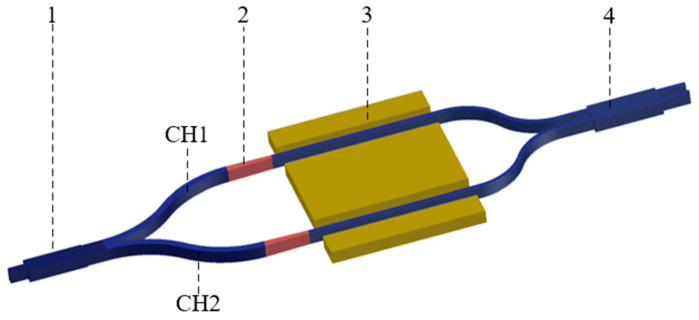
Schematic of the designed interferometer: 1: beam splitter; 2: mode converter; 3: electrode; 4: directional coupler.

The schematic diagram of the proposed polarization-insensitive LN interferometer is shown in Figure 1. The light is injected from the input waveguide and divided into two modulation waveguides after passing through the optical beam splitter. The two mode converters are integrated at the front and the back of the modulation waveguides, respectively. After passing through the front mode converter, the TE_0_ mode maintains its polarization without mode conversion and is modulated directly, while the TM_0_ mode is converted to the TE_1_ mode, guaranteeing the subsequent modulation. The TE_1_ (converted from the TM_0_ mode) and initial TE_0_ mode are modulated by the electrical signal in the modulation regions, then the lights are converted to the same mode as the input lights, and finally the lights are combined in the 2 × 2 MMI. Meanwhile, the modulation regions use the reported structure with the voltage–length product of 2.2 Vcm and an insertion loss of 2.5 dB [24]. Due to limited equipment and computing power, this study only simulated each device separately and then evaluated the overall device after obtaining the results.

### 2.1. Mode Converter

**Figure 2:**
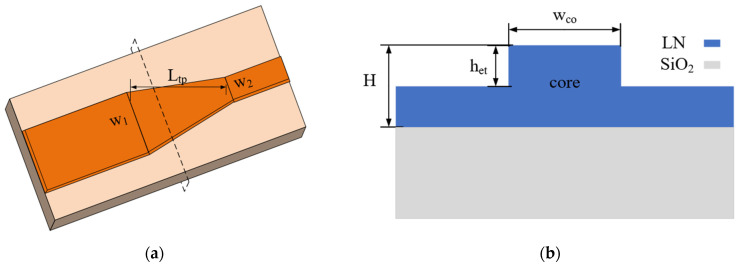
(**a**) Schematic diagram of mode converter. (**b**) Cross-section of the waveguide.

The mode converter is the key component of a polarization-independent interferometer. It was designed based on mode hybridization in the waveguide taper, with a variable waveguide width. The employed taper structure is shown in Figure 2a. This structure is a conventional lateral taper in which the waveguide width varies while maintaining a constant etch depth. Due to its simplicity in design and fabrication, regular lateral tapers are commonly used to adjust the lateral dimensions of waveguide modes [25]. In an LN waveguide, mode conversion between TM_0_ and TE_1_ modes, which is judged based on intensity distribution [26,27], is expected to occur as light propagates through the taper structure.

**Figure 3:**
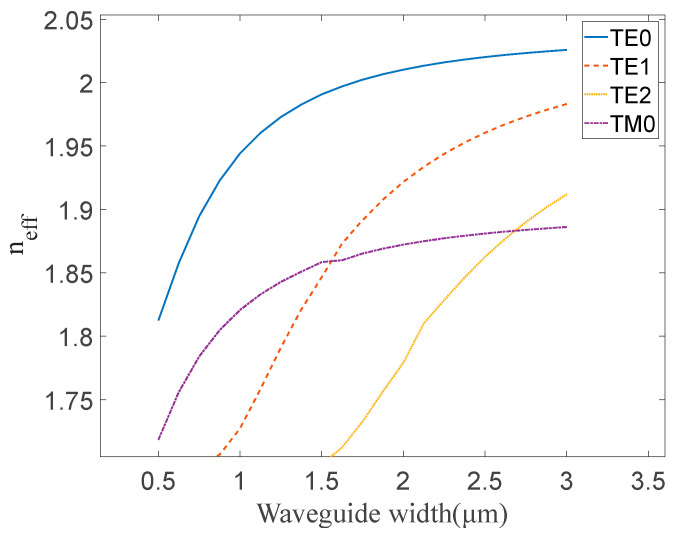
The calculated effective refractive index for the eigenmodes with different widths.

We analyzed the modes using the finite-difference eigenmode (FDE) method in the waveguide, with different widths between 0.5 μm and 3 μm used to characterize mode transmissions in tapered waveguides, and the results are shown in Figure 3.

**Figure 4:**
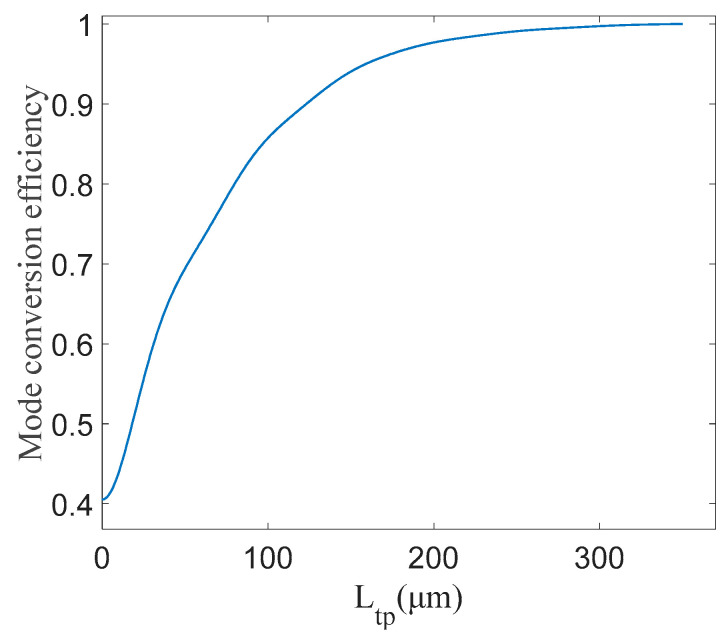
Relation between taper structure length and conversion efficiency.

In Figure 3, the effective refractive index of the LN waveguide is shown for an etch depth (h_et_) of 0.4 μm and a total height (H) of 0.6 μm while the core width (w_co_) increases from 0.5 μm to 3 μm. Due to the vertical asymmetry of the waveguide, mode conversion phenomena were predicted to occur in certain width ranges. As shown in Figure 3, the effective refractive index curves of TM_0_ and TE_1_ intersect at approximately w_co_ = 1.6 μm, where mode conversion occurs. This conversion is achieved when the waveguide width (w_1_, w_2_) satisfies the condition w_1_ < w_co_ < w_2_, which is used to perform the polarization rotation. Therefore, the waveguide width of the taper region is chosen to decrease from 2 μm to 1.4 μm, and the corresponding mode conversion efficiency is estimated. As shown in Figure 4, using the eigenmode expansion (EME) method, high mode conversion efficiency (>90%) can be achieved when the length of the conversion region is longer than 125 μm. Mode conversion efficiency measures the ratio of the energy of the goal mode to the input mode. Since the mode converter is reversible, mode conversion efficiency refers to the efficiency of the conversion between TE_1_ and TM_0_ modes; meanwhile, the light mode can be judged by the intensity distribution.

**Figure 5:**
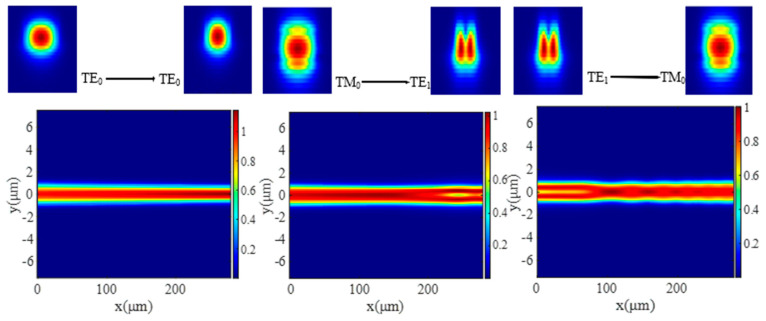
Schematics of TE_0_-TE_0_, TM_0_-TE_1_, and TE_1_-TM_0_ mode converters with corresponding mode profiles.

Utilizing the 3D finite-difference time-domain (FDTD) method, the results of light mode conversion with TE_0_, TM_0,_ and TE_1_ input modes are shown in Figure 5, respectively. It shows that the input TE_0_ mode does not undergo mode conversion, while the other two input modes experience a remarkable mode conversion.

### 2.2. Multimode Interference Coupler

The operational principle of an MMI coupler is based on the self-imaging phenomenon in multimode waveguides. This phenomenon results in the periodic reproduction of the input field distribution as one or more images. Therefore, a fundamental prerequisite for achieving the MMI effects is a waveguide capable of supporting multiple modes. In the case of ridge waveguides, sufficiently wide waveguides can support a variety of modes. The wider the waveguide is, the larger the number of modes it can support, leading to the enhancement of image quality. However, it is worth noting that a wider waveguide requires a longer MMI region.

In the proposed interferometer, two kinds of MMIs are designed, i.e., a 1 × 2 MMI and a 2 × 2 MMI are designed at the input and output ends, respectively. The 1 × 2 MMI is used as a beam splitter device, while the 2 × 2 MMI is used as a directional coupler. The 1 × 2 MMI distributes the input light energy into both arms of the splitter equally, while the 2 × 2 MMI ensures uniform distribution of light input from one arm to both output arms in the directional coupler. By designing the width of the 2 × 2 MMI, MMIs can satisfy various requirements, for example, near-zero anomalous group-velocity dispersion for photon-pair generation, optical switch [1,10,28], and 3 dB beam splitter [29]. However, this study concerns the transmission of two polarizations.

**Figure 6:**
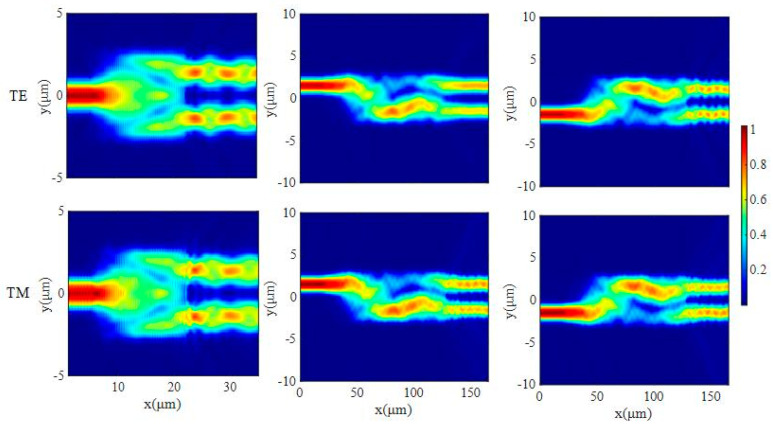
The 1 × 2 MMI and 2 × 2 MMI electric field evolution diagram.

Simulations of the transmission of TE_0_ and TM_0_ modes were conducted using the 3D FDTD method. Figure 6 shows the propagation of TE_0_ and TM_0_ modes in the 1 × 2 MMI and 2 × 2 MMI, respectively. It shows that the 1 × 2 MMI can evenly split the input light energy and transfer it to two output ports, whether it is the TE_0_ or TM_0_ mode. Furthermore, the input light from any input port of the 2 × 2 MMI can be evenly split into two output ports on the condition of TE_0_ and TM_0_ modes indicating that the 2 × 2 MMI can realize beam splitting for TE_0_ and TM_0_.

Defining $L_{\pi }$ as the beat length of the two lowest-order modes, it can be described by

(1) $$L_{\pi }=\frac{\pi }{\beta_{0}-\beta_{1}}=\frac{4n_{r}{W_{e}}^{2}}{3\lambda_{0}}$$

where *W_e_* is the effective waveguide width in the multimode interference region, *n_r_* is the core refractive index, the incident wavelength in free space is *λ*_0_, and the propagation constant of order *v* is $\beta_{v}$ and can be described as

(2) $$\beta_{v}=\frac{2\pi n_{v}}{\lambda }$$

where $n_{v}$ is the effective refractive index of the *v* mode.

According to different incident conditions, an MMI can be divided into three types: ordinary interference, symmetrical interference, and paired interference. The three types have different positions for the first N-fold, which are $3L_{\pi }/N$, $3L_{\pi }/4N$, and $L_{\pi }/N$, respectively.

In order to reduce loss, a conical waveguide is used at the connection of the multimode waveguide region. Additionally, the 1 × 2 MMI is selected to be the symmetric interference type while the 2 × 2 MMI is selected to be the ordinary interference type to reduce the footprint of the interferometers.

The 1 × 2 MMI multimode waveguide width is preliminarily set to be 5.2 μm. The shortest length of the 1 × 2 MMI coupler is $L_{MMI}=3L_{\pi }/8$. When $W_{MMI}=5.2$ μm, $n_{0}$ and $n_{1}$ are found to be 2.035 and 2.021 by calculating that $\beta_{0}$ and $\beta_{1}$ are 8.251 and 8.194, respectively. The length of the $L_{\pi }$ is 55.11 μm and the length of the MMI is calculated as $L_{MMI}=20.67$ μm. It is a rough initial value, and it is necessary to scan around the value to determine the exact value.

**Figure 7:**
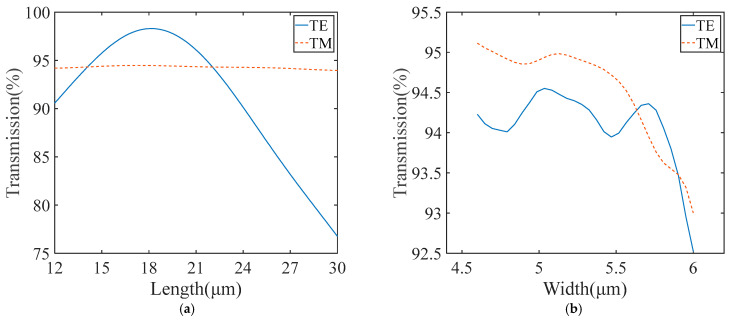

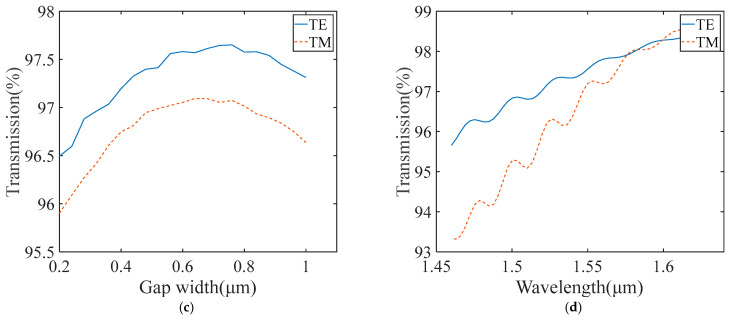
(**a**) The transmission of the 1 × 2 MMI with different length. (**b**) The transmission of the 1 × 2 MMI with different width. (**c**) The transmission of the 1 × 2 MMI with different gap width. (**d**) The transmission of the 1 × 2 MMI with different wavelength.

We utilize the 3D FDTD method to simulate light evolution in the MMI with different widths and lengths. As shown in Figure 7a, when the length of the MMI is 17 μm, the width of the MMI is 5.2 μm, and the gap width is 0.5 μm, the transmissions of both modes reach a maximum, which is more than 94.45%. When the length is between 14 and 22 μm, the MMI has a good transmission result. As shown in Figure 7b, the transmission of the MMI was calculated with the width varying from 4.5 μm to 6 μm, with the length of MMI as 17 μm, and the gap width (the distance between two single-mode waveguides at MMI input/output) as 0.5 μm. The results show that the transmissions of TE_0_ and TM_0_ modes both exceed 94.4% when the MMI width is 5.2 μm. In addition, the transmission results of the two modes are still over 94% with the MMI width between 4.5 μm and 5.8 μm. Figure 7c shows the transmission versus gap width, with 5.2 μm width and 17 μm length of the MMI. The highest transmission is obtained when the gap width reaches 0.76 μm. Since the gap width determines the transverse positions of two single-mode waveguides, the highest transmission is achieved when the fundamental mode profile in two single-mode waveguides matches well with the first-order mode profile in MMI. From the above discussion, it can be concluded that this MMI has a good fabrication tolerance. Additionally, Figure 7d shows the transmission versus wavelength, where the highest value takes place at 1.6 μm. Meanwhile, the transmissions at all concerned wavelengths exceed 93.4%, demonstrating that the 1 dB bandwidth of the beam splitter includes C, L, and S telecommunication bands ranging from 1460 nm to 1625 nm.

Based on the design of the 1 × 2 MMI, the 2 × 2 MMI was designed using the same method. The multimode waveguide width of the 2 × 2 MMI is preliminarily set to 6 μm. When $W_{MMI}=6$ μm, $n_{0}$ and $n_{1}$ are 2.038 and 2.026, as determined by calculating that $\beta_{0}$ and $\beta_{1}$ are 8.260 and 8.214, respectively, the length of $L_{\pi }$ is 68.29 μm, and the MMI is calculated as $L_{MMI}=102.44$ μm.

**Figure 8:**
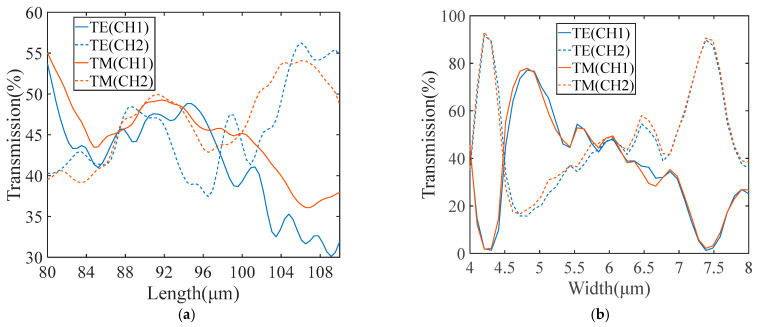
(**a**) The transmission of the 2 × 2 MMI with different length. (**b**) The transmission of the 2 × 2 MMI with different width.

As shown in Figure 8a, when the length of the MMI is 92 μm, the transmissions of both channels and modes reach a maximum, which is more than 47%. Meanwhile, the two transmissions of the outputs are approximately equal. Figure 8b shows the transmission curves of the 2 × 2 MMI with the width varying from 4 μm to 8 μm, seen on the condition of a 92 μm length of the MMI. When the MMI width is 6 μm, the transmissions of both channels and modes reach 47%. In addition, the transmissions of both channels and modes continue to exceed 45% with the MMI width between 5.85 μm and 6.15 μm. Since the MMI length heavily determines the transmission and beam-splitting ratio of the directional coupler, it impacts the interference visibility of the proposed interferometer. Therefore, we calculate the transmission, with the MMI length varying from 80 μm to 110 μm and with the width of the MMI as 6 μm.

**Figure 9:**
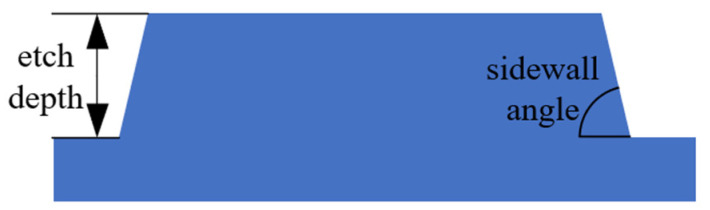
A schematic diagram of the sidewall angle and etching depth.

**Figure 10:**
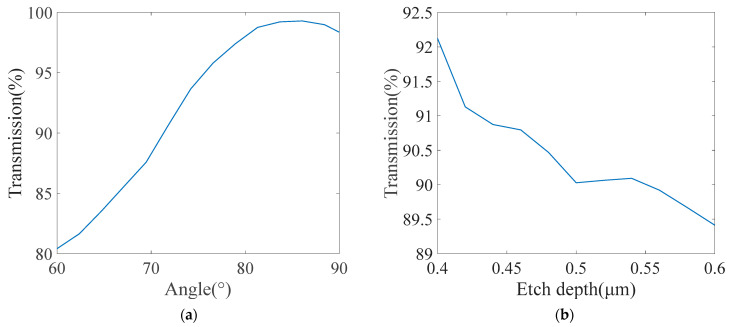
(**a**) The transmission of the 2 × 2 MMI with different inclination angle. (**b**) The transmission of the 2 × 2 MMI with different etch depth.

Figure 9 illustrates the definition of etching depth and sidewall angle. Figure 10a shows the 2 × 2 MMI transmission curves of TE_0_ mode, with the sidewall angle varying from 60° to 90°, on the condition of a 6 μm width, 92 μm length, and 1 μm gap width. We set the upper surface width of the ridge waveguide to be invariable, so the cross-sectional area of the LN waveguide is reduced with the increase in inclination angle. The cross-sectional area varies with the inclination angle, which affects the effective refractive index; therefore, the smaller the inclination angle, the lower the transmission will be. When the angle is between 75° and 90°, the MMI has a good transmission result that exceeds 95%. With the geometry parameters unchanged, Figure 10b shows the 2 × 2 MMI transmission curves of the TE_0_ mode with the etch depth varying from 0.4 to 0.6 μm. The effective refractive index is also affected by the etch depth, and so the higher the etch depth is, the lower the transmission will be. The MMI show a transmission result over 90% when the etch depth is between 0.4 and 0.5 μm. It can be concluded that this MMI has a good fabrication tolerance in terms of the inclination angle and the etch depth.

## 3. Performance of the Designed Polarization-Independent Interferometer

The TE_0_ and TM_0_ modes are injected into the input end of the beam splitter, which divides the input beam into two arms. Passing through the following mode converter, the input TE_0_ and TM_0_ modes become TE_0_ and TE_1_ modes, respectively. Then, the converted modes enter the modulation arms, one of which is imposed on a gradually increased phase shift.

**Figure 11:**
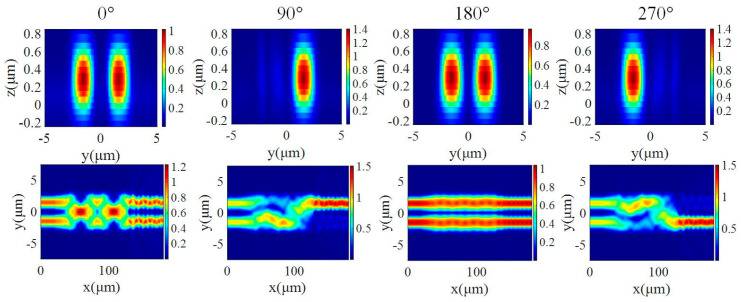
The output mode profile and energy distribution in the 2 × 2 MMI for TE polarization with respect to different phase shifts.

For the input TE_0_ mode, at 1550 nm, the transmission of the two arms at the output end was calculated, and the results are shown in Figure 11. When the two waveguides are in phase, the transmission of the output end is 49.4%. When the phase of the two waveguides is orthogonal, the transmission of the output end is 1.2% and 98.3%, respectively. The extinction ratio is calculated to be as high as 97.95%.

**Figure 12:**
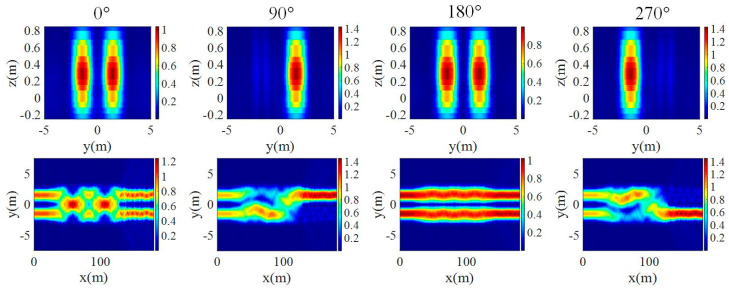
The output mode profile and energy distribution in the 2 × 2 MMI for TM polarization with respect to different phase shifts.

When the input is TM_0_ mode at 1550 nm, the transmission of the two arms at the output end is simulated as shown in Figure 12. The transmission of the output end is 48.2% when the two waveguides are in phase. On the condition of orthogonal phase, the transmission of the output end is 0.88% and 94.95%, respectively. The extinction ratio is calculated as 98.16%, which is a little lower than that of the TE_0_ mode.

**Figure 13:**
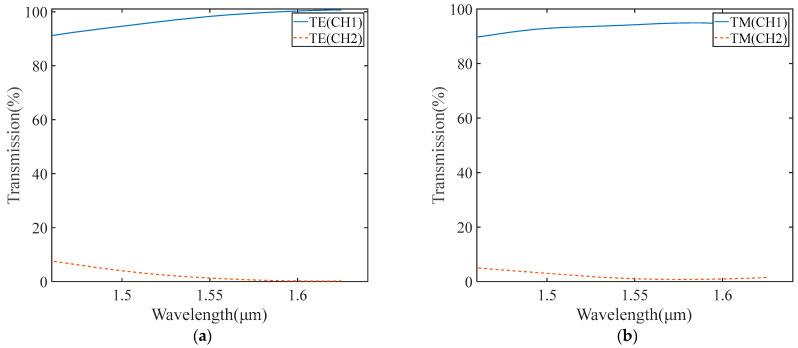
(**a**) The work bandwidths for TE. (**b**) The work bandwidths for the TM mode.

The work bandwidth for TE and TM modes was researched. The transmissions of two outputs were calculated with varying wavelengths in an orthogonal phase condition, as shown in Figure 13. Where the lowest extinction ratio takes place at 1.46 μm, the transmissions of TE are 91.16% and 7.6%, and the transmissions of TM are 89.65% and 5.1%, respectively. Therefore, the extinction ratio is more than 85%, demonstrating that the 1 dB bandwidth of the beam splitter includes C, L, and S telecommunication bands ranging from 1460 nm to 1625 nm.

## 4. Conclusions

We propose a polarization-independent EO interferometer based on an X-CUT LN platform. This innovative design offers a promising solution for overcoming the polarization dependency typically associated with the LN interferometer. The geometric structures of the splitter and directional coupler are carefully designed within the telecommunication bands, while losses are kept at a low level. Furthermore, it exhibits significant tolerance to size variations, decreasing the pressure of fabrication. At 1550 nm, we numerically demonstrated that the extinction ratios of 97.59% and 98.16% were achieved for TE and TM modes, respectively. The proposed interferometer had prospective applications in efficient optical control, optical telecommunications, and quantum information processing owing to its simplicity in design, ease of fabrication, and ability to accommodate both polarizations.

## Data Availability

The original contributions presented in the study are included in the article. Further inquiries can be directed to the corresponding author.

## References

[1] Comellas J., Junyent G. In Optical Interconnection for Datacenters: To Switch or Not to Switch. Proceedings of the 2023 23rd International Conference on Transparent Optical Networks (ICTON).

[2] Goodman J.W., Leonberger F.J., Sun-Yuan K., Athale R.A. (1984). Optical interconnections for VLSI systems. Proc. IEEE.

[3] Haurylau M., Chen G., Chen H., Zhang J., Nelson N.A., Albonesi D.H., Friedman E.G., Fauchet P.M. (2006). On-Chip Optical Interconnect Roadmap: Challenges and Critical Directions. IEEE J. Sel. Top. Quantum Electron..

[4] Kahn J., Kazovsky L. (2006). Coherent Optical Communications: Fundamentals and Future Prospects, Frontiers in Optics, Rochester, New York, 2006/10/10.

[5] Miller D.A.B. (2009). Device Requirements for Optical Interconnects to Silicon Chips. Proc. IEEE.

[6] Reed G.T., Mashanovich G., Gardes F.Y., Thomson D.J. (2010). Silicon optical modulators. Nat. Photon..

[7] Shinya A., Kida K., Sato H., Lu G.W., Yokoyama S., Fujikata J. In High-Speed Optical Convolutional Neural Network Accelerator with 100 Gbaud EO-polymer/Si Hybrid Optical Modulator. Proceedings of the 2023 Opto-Electronics and Communications Conference (OECC).

[8] Xu Q., Schmidt B., Pradhan S., Lipson M. (2005). Micrometre-scale silicon electro-optic modulator. Nature.

[9] Gupta S., Sharma S., Ahmad T., Kaushik A.S., Jha P.K., Gupta V., Tomar M. (2021). Demonstration of efficient SBN thin film based miniaturized Mach Zehnder EO modulator. Mater. Chem. Phys..

[10] Li C., Zheng W., Dang P., Zheng C., Zhang D. (2017). Investigation of a low-voltage polymeric 2×2 Mach-Zehnder interferometer optical switch using five-serial-coupled electro-optic microrings. Optik.

[11] Thomas R., Ikonic Z., Kelsall R.W. (2012). Electro-optic metal–insulator–semiconductor–insulator–metal Mach-Zehnder plasmonic modulator. Photon. Nanostruct. Fundam. Appl..

[12] Kieninger C., Kutuvantavida Y., Elder D.L., Wolf S., Zwickel H., Blaicher M., Kemal J.N., Lauermann M., Randel S., Freude W. (2018). Ultra-high electro-optic activity demonstrated in a silicon-organic hybrid modulator. Optica.

[13] Tang Y., Peters J.D., Bowers J.E. (2012). Over 67 GHz bandwidth hybrid silicon electroabsorption modulator with asymmetric segmented electrode for 1.3 μm transmission. Opt. Express.

[14] Bazzan M., Sada C. (2015). Optical waveguides in lithium niobate: Recent developments and applications. Appl. Phys. Rev..

[15] Li X., Zhao Y., Tao J., Li R., Liu J., Li J. (2024). Linearity-Enhanced integrated lithium niobate modulator based on carrier separated asymmetric Mach−Zehnder structure. Opt. Laser Technol..

[16] Albota M.A., Wong F.N.C., Shapiro J.H. (2006). Polarization-independent frequency conversion for quantum optical communication. J. Opt. Soc. Am. B.

[17] Jignesh J., Corcoran B., Schröder J., Lowery A. (2017). Polarization independent optical injection locking for carrier recovery in optical communication systems. Opt. Express.

[18] Chen D., Zhang X., Fan K., Wang J., Lu H., Wang Q., Wu S., Hao R., Li Z., Jin J. (2023). Experimental demonstration of a hybrid OFDMA/NOMA scheme for multi-user underwater wireless optical communication systems. Opt. Commun..

[19] Saxena A. (2023). On the role of optical materials in the realization of schemes for secure quantum communication. Mater. Today Proc..

[20] Pan S., Zhang Y. (2012). Tunable and wideband microwave photonic phase shifter based on a single-sideband polarization modulator and a polarizer. Opt. Lett..

[21] Ganjali M., Esmail Hosseini S. (2019). Effects of frequency chirping and finite extinction ratio of optical modulators in microwave photonic IFM receivers. Opt. Commun..

[22] Hsu C.W., Huang C.F., Tsai W.S., Wang W.S. (2017). Lithium Niobate Polarization-Independent Modulator Using Integrated Polarization Splitters and Mode Converters. J. Lightw. Technol..

[23] Song L., Liu W., Guo Z., Li H., Xie Y., Yu Z., Li H., Shi Y., Dai D. (2023). Anisotropic Thermo-Optic Mach–Zehnder Interferometer on LNOI for Polarization Handling and Multiplexing. Laser Photon. Rev..

[24] He M., Xu M., Ren Y., Jian J., Ruan Z., Xu Y., Gao S., Sun S., Wen X., Zhou L. (2019). High-performance hybrid silicon and lithium niobate Mach–Zehnder modulators for 100 Gbit s−1 and beyond. Nat. Photon..

[25] Dai D., Tang Y., Bowers J.E. (2012). Mode conversion in tapered submicron silicon ridge optical waveguides. Opt. Express.

[26] Daoxin D., Sailing H., Hon-Ki T. (2006). Bilevel mode converter between a silicon nanowire waveguide and a larger waveguide. J. Lightw. Technol..

[27] Zhang J., Qiu P., He R., Song X., Dai Z., Liu Y., Pan D., Yang J., Guo K. (2024). Compact mode converters in thin-film lithium niobate integrated platforms. Opt. Lett..

[28] Calò G., Bellanca G., Fuschini F., Barbiroli M., Bertozzi D., Tralli V., Petruzzelli V. (2023). 4 × 4 Integrated Switches Based on On-Chip Wireless Connection through Optical Phased Arrays Photonics. Photonics.

[29] Liu X., Zhao Y., Zhu Z., Liu H., Gan F. (2023). Particle Swarm Optimized Compact, Low Loss 3-dB Power Splitter Enabled by Silicon Columns in Silicon-on-Insulator Photonics. Photonics.

